# Two birds, one stone: a surgical approach for third nerve palsy with aberrant regeneration

**DOI:** 10.5935/0004-2749.20220065

**Published:** 2025-08-22

**Authors:** Ana Letícia Fornazieri Darcie, Iara Debert, Mariza Polati

**Affiliations:** 1 Department of Ophthalmology, Hospital das Clínicas, Faculdade de Medicina, Universidade de São Paulo, São Paulo, SP, Brazil

**Keywords:** Oculomotor nerve diseases/surgery, Strabismus, Blepharoptosis, Eye movement/physiology, Ophthalmologic sur gical procedure, Nerve regeneration, Human, Case report, Doença do nervo oculomotor/cirurgia, Estrabismo, Blefaroptose, Movimento ocular/fisiologia, Procedimento cirúrgico oftalmológico, Regeneração nervosa, Humanos, Relato de caso

## Abstract

Aberrant regeneration in third nerve palsies, linking medial rectus contraction to the levator palpebrae muscle, is a great opportunity for surgical planning to address both the ptosis and horizontal deviation in a single procedure. We report a case of severe ptosis associated with exotropia that was successfully corrected with a single horizontal strabismus surgery owing to aberrant regeneration and discuss the basis underlying the surgical planning.

## Abstract (pt)

A regeneração aberrante nas paralisias do terceiro nervo, ligando a contração do reto medial ao músculo levantador da pálpebra, é uma grande oportunidade para fazer um planejamento cirúrgico para tratar tanto a ptose quanto o desvio horizontal em um procedimento único. Relatamos uma ptose grave associada à exotropia corrigida com sucesso com uma única cirurgia de estrabismo horizontal devido à regeneração aberrante e discutimos as bases do planejamento cirúrgico.

## INTRODUCTION

Surgical management of third nerve palsy is challenging. Ptosis repair in these patients is traditionally recommended with undercorrection to avoid exposure keratitis due to the poor Bell’s phenomenon. Consequently, multiple surgical procedures are often required to achieve a satisfactory result^(1-3)^. Aberrant regeneration is a synkinesis miswiring caused by a disruption of the endoneural integrity, with retrograde degeneration of the damaged axon, followed by peripheral misdirection of the regenerating axons. The most common feature of synkinesis miswiring in third nerve palsy is the medial rectus muscle fibers misdirected to the levator muscle; that is, the upper eyelid is ptotic in the primary position but elevates when the eye is adducted^(4)^. Even though the presence of aberrant regeneration adds more complexity to the clinical setting^(5)^, judicious use of this phenomenon may correct the ptosis, thereby obviating the need of eyelid surgery^(6)^.

O’Donnell et al.^(7)^ were the first to show how aberrant regeneration could be used to correct ptosis by increasing innervation to the medial rectus muscle. They described a recess-resect surgical approach for the horizontal muscles of the healthy eye to correct both exotropia and ptosis in a single surgical procedure. A few reports on a similar approach have been published in the literature since then^(5,6,8)^, but it was only more recently that Fouad and coworkers published the largest series with 11 cases^(9)^. Only 3 patients presented with severe ptosis, and all of them showed postoperative ptosis ranging from 1.5 to 2.0 mm.

Herein, we report a case of third nerve palsy with severe ptosis and aberrant regeneration that was treated with horizontal strabismus surgery to address both the ptosis and ocular misalignment. To our knowledge, this is the first reported case of severe ptosis totally corrected with a single strabismus surgery. We also discuss the innervational basis underlying the surgical plan.

## CASE REPORT

**Figure 1:**
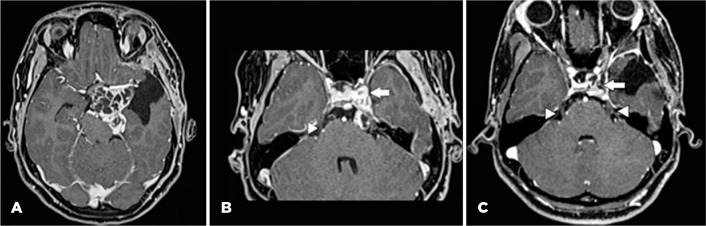
(A) Preoperative brain magnetic resonance image showing an expansive lesion suggestive of left trigeminal nerve schwannoma involving the interpeduncular, suprachiasmatic, optic-carotid, and ambiens cisterns and the area of previous resection in the left temporal operculum, amygdala, and part of the hippocampus. (B) Magnetic resonance image showing the origin of the lesion in the left trigeminal nerve in its cisternal portion (arrowhead indicating the preserved right trigeminal nerve) and the anterior extension of the lesion anterior to the entrance of the cavernous sinus (arrow). (C) Postoperative magnetic resonance image demonstrating the subtotal resection of the lesion with residual tumor involving the cavernous sinus and Meckel’s cave (arrow). The arrowhead indicates the visualization of both trigeminal nerves in their cisternal parts. No orbital findings can be observed.

An 18-year-old female patient presented with left ptosis and exotropia after resection of a cavernous sinus schwannoma 15 months previously. Figure 1 (A and B) shows the lesion when she was referred to our service, after a prior surgery in another hospital. Figure 1C shows the postoperative aspect. Her past ocular history was unremarkable. Cranial nerve assessment revealed hypoesthesia in the left hemiface without facial weakness.

**Figure 2:**
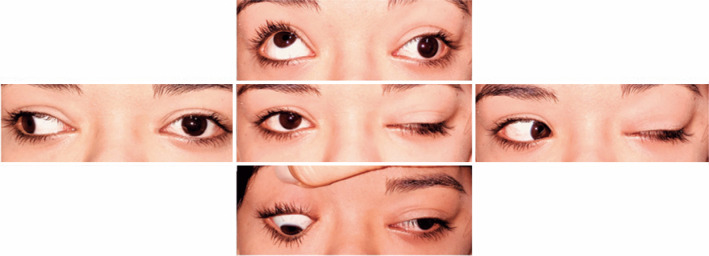
Preoperative motility assessment. Noticeable left deficits of adduction, elevation, and depression with the left upper eyelid elevation were observed during the attempted left adduction and downgaze.

**Figure 3:**
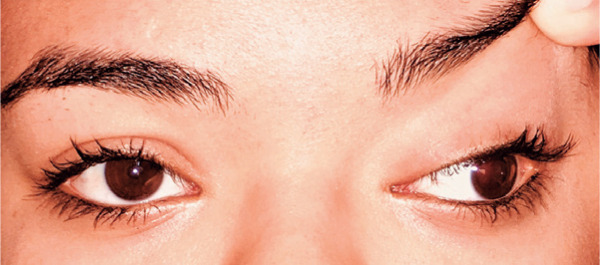
Preoperative deviation in primary position with the left upper eyelid lifted.

Her distance visual acuity was 1.0 OU and near visual acuity was OD J1/OS J4. Severe left eyelid ptosis and Bell’s phenomenon were absent in the left eye. The anterior and posterior segment evaluation was unremarkable except for mydriasis with no light response in the left eye. Ductions were full in the right eye, and marked deficits (-4) of adduction, elevation, and depression were found in the left eye. The left upper eyelid elevation was appreciated during attempted left eye adduction and downgaze (Figure 2). Both distance and near deviation were used to measure exotropia 60∆ using the Krimsky test. No vertical deviation was observed in the primary position (Figure 3). The patient could achieve fusion with horizontal prisms, and no torsion was observed in the Maddox test. Forced duction testing revealed mild limitation of left eye adduction. In the active force generation testing, a mild active force was observed in the left medial rectus muscle, so a diagnosis of incomplete left third nerve palsy with aberrant regeneration was made.

We decided to take advantage of the aberrant regeneration phenomenon to address both the strabismus and ptosis in the same surgical procedure. Considering the paretic pattern of the deviation, a recess-resect procedure was also performed on the left eye. A right lateral rectus recession of 9 mm, right medial rectus resection of 5 mm, left lateral rectus recession of 10 mm, and left medial rectus resection of 9 mm were planned.

**Figure 4:**
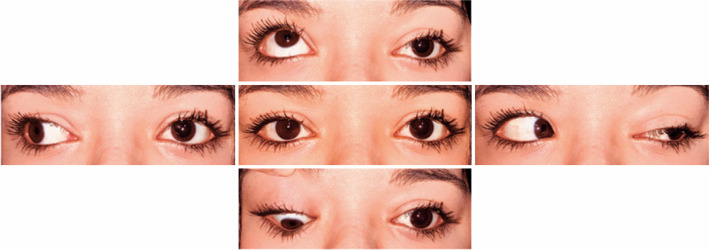
One-year postoperative assessment. The patient was orthotropic in the primary position.

The surgical procedure was uneventful. Four weeks after the operation, the patient was orthotropic, and no ptosis was observed in the primary position. Narrowing of the palpebral fissure on abduction and slight widening on adduction of the left eye were observed. Ductions remained full in the right eye. Persistent left eye deficits of upgaze and downgaze (-4), improved left eye adduction (-3), and mild left eye abduction deficit (-2) were found (Figure 4). One year after the surgery, the measurements remained stable. The patient regained high-grade stereopsis (60 arc-sec) and remained without torsion.

## DISCUSSION

Third nerve palsy is one of the leading postoperative ophthalmological complications of cavernous sinus surgery and can present with aberrant regeneration. The most frequently observed clinical manifestation is elevation of the upper lid on attempted adduction (inverse Duane’s sign) or downgaze (Pseudo-Von Graefe’s sign)^(10)^. Our patient showed both signs.

Aberrant regeneration, causing upper eyelid elevation on attempted adduction, provides an opportunity to correct the ptosis through horizontal strabismus surgery^(6)^. According to Hering’s law, by forcing the unaffected fixating eye to an adduction position and therefore causing constant abduction innervation, the medial rectus muscle of the affected eye is also stimulated, and the eyelid raises^(5)^. Performing a modified Kestenbaum reposition operation may create fixation duress on the affected eye medial rectus muscle, thereby correcting the ptosis in primary gaze with no eyelid surgery. The horizontal deviation can be addressed in the non-fixating eye by combining the surgical dosage for the strabismus procedure and applying it to the modified Kestenbaum operation. Depending on the magnitude of the misalignment, one or both eyes may require operation.

Most cases reported in the literature had surgery only on the non-deviating eye. Conversely, in our case, both eyes were treated with surgery because the angle deviation was large and our aim was to avoid submaximal surgery on the healthy eye with possible duction limitation postoperatively. The case reported herein differs from those reported in the previous studies in another significant point, which is the successful correction of severe ptosis in our patient that was achieved with a single procedure. Gottlob et al.^(5)^ reported the use of horizontal surgery to address both the ptosis and exotropia in two patients. In one patient, three surgeries were necessary to achieve good alignment in the primary position and good eyelid position. They also performed the Scott superior oblique procedure to accomplish good horizontal alignment. Nguyen addressed cases of moderate ptosis treated with a large lateral recess and small medial rectus resection in the non-involved eye with adjustable sutures^(6)^. In the case series of Fouad et al.^(9)^, only four patients showed no ptosis after surgery, and all of them presented with small-to-moderate ptosis preoperatively (2-4 mm). The patients with severe ptosis still showed some degree of ptosis postoperatively. The apparently favorable surgical result was not related to the severity of the ptosis but to the eyelid position during the attempted adduction.

This case highlights the value of a detailed preoperative assessment for identifying signs of synkinetic miswiring in third nerve palsy. The judicious use of this phenomenon in appropriate patients allows the correction of severe ptosis and large angle exotropia in a single, effective procedure.
